# The effect of Malaysia general election on stock market returns

**DOI:** 10.1186/s40064-016-3648-5

**Published:** 2016-11-14

**Authors:** Venus Khim-Sen Liew, Racquel Rowland

**Affiliations:** Centre of Excellence for Business, Economics and Finance Forecasting, Faculty of Economics and Business, Universiti Malaysia Sarawak, 94300 Kota Samarahan, Sarawak Malaysia

**Keywords:** Stock market, Malaysia, General election effect, G14

## Abstract

During the latest episode of general election held in Malaysia, it is observed that the FBMKLCI index was lifted 62.52 points in a day soon after the announcement of election outcome. Moreover, the index registered a highest gain of 96.29 points in the middle of the intra-day trade. This suggests that investors who had got the right direction could make profitable intra-day trading the next trading day of the general election date. Results from statistical analysis uncover significant before-election-effect and after-election-effect from the most recent general elections held in Malaysia. Different subsets of macroeconomic variables are found to have significant role on stock market return depending on the market situation. Remarkably, when there was close fight between the two major political parties during the 2008 and 2013 election years, political uncertainty showed up its negative and significant role in influencing the stock market return. The major implication of these findings is that while investors may seek abnormal returns before and after the next general election, which is around the corner, they will have to pay attention on the influence of macroeconomic variables and political uncertainty on stock market return during the election year.

## Background

Academics from the financial economics stream and researchers of investment institutions are dedicated to uncover market anomaly, if any, in stock markets. Among other market anomalies, political election effect has attracted their continuous attention. Several researchers have put forward their arguments to postulate that political election could have significant impact on stock market performance. For instance, it is argued that incumbents tend to stimulate the economy condition to re-election and to pursue deflationary policies afterwards (Nordhaus 1975). In similar point of view, Ragoff (1990) suggests the equilibrium political budget cycle which asserts that incumbent government tends to bias pre-election fiscal policy.

From another perspective, Hibbs (1977) proposes the partisan theory which presents a reveal preference of political parties toward various economic policies. According to Hibbs (1977), labor-oriented parties tend to focus on employment rather than inflation, while business-oriented parties focus on price stability rather than to unemployment. Hence, it can be hypothesized that political elections will have significant effect on the stock market which reflects the economic performance. See *inter alia* Allvine and O’Neill (1980), Worthington (2006), Floros (2008), Abidin et al. (2010), for empirical evidences supporting the hypothesis.^1^ For a preview, Allvine and O’Neill (1980) reports that the US stock market had a rising trend over the two years prior to the Unites State’s presidential elections. Meanwhile, Worthington (2006) uncovers that stock returns perform better under Liberal-National than Labour ministries the Australian stock market. From the general election point of view, Floros (2008) documents a significant impact of general election on the course of Athen Stock Exchange (ASE). Besides, Abidin et al. (2010) find that the returns of New Zealand Exchange are significantly higher during the election in year 2002.

It is noteworthy that there is another strand of interesting research on political elections, stock market volatility, and stock market performance (see among others, Bialkowski et al. 2008; Goodell and Vähämaa 2013; Johnson et al. 1999; Kirui et al. 2014; Kabiru et al. 2015; Lehkonen and Heimonen 2015; Li and Born 2006; Opare 2012; Smales 2014, 2015, 2016). In particular, Bialkowski et al. (2008) found evidence that stock market volatility is substantially raised around national elections over 27 industrialised nations. Smales (2014, 2016) documented that the implied volatility of financial markets increases in line with uncertainty about the election outcome. Morover, Li and Born (2006) and Goodell and Vähämaa (2013) found that stock market volatility rises when the US presidential election does not have an obvious winner, while Smales (2015) reported increasing likelihood of the incumbent party winning reduces stock market uncertainty. Smales (2014, 2016) documented that the implied volatility of financial markets increases in line with uncertainty about the Australia election outcome.

On the other hand, Pastor and Veronesi (2012, 2013) provided the theoretical discussion on how political uncertainty could have impacts on market prices. Meanwhile, Lehkonen and Heimonen (2015) provided evidence that political uncertainty had significant impact on stock market performance of 49 emerging markets.

The current study analyses the effect of general election on the Malaysia stock market. There have been thirteen general elections so far ever since Federation of Malaya received its independence in 1957.^2^ In Malaysia, the National Front and the People’s Alliance are the two major political parties participating in general election. The National Front coalition has been in power throughout the whole episodes of Malaysia’s general election, although in the recent few episodes the opposition had given the former fierce challenges. Table 1 presents a comparison of general election between government and opposition that covers from 1959 to 2013. It is obviously that National Front has dominated the seats of House of Representatives in every general election as well as becoming the federal government for the past 57 years. Note that during the sample period of the current study, the percentage vote for the opposition is far behind the incumbent government for 1995, 1999 and 2004. However, the opposition had given fierce challenge to the incumbent in the two most recent episodes of general election. Nonetheless, the results gained from every election are likely to be inconsistent although National Front holds majority of the seats. For example, National Front won 59.91% seats with 46.53% votes whereas People’s Alliance only won 40.09% seats with 53.47% votes in the last election.

**Table 1:** Comparison of general election between government and opposition from 1959 to 2013 *Source*: Election Commission of Malaysia (2016)

Year	Government			Opposition			Total seats
	Seats	% seats	% vote	Seats	% seats	% vote	
1959	74	71.15	51.70	30	28.85	48.30	104
1964	89	85.58	58.50	15	14.42	41.50	104
1969	95	65.97	49.30	49	34.03	50.70	144
1974	135	87.66	60.70	19	12.34	39.30	154
1978	130	84.42	57.20	24	15.58	42.80	154
1982	132	85.71	60.50	22	14.29	39.50	154
1986	148	83.62	55.80	29	16.38	44.20	177
1990	127	70.56	53.40	53	29.44	46.60	180
1995	162	84.38	65.20	30	15.63	34.80	192
1999	148	76.68	56.50	45	23.32	43.50	193
2004	198	90.41	63.90	21	9.59	36.10	219
2008	140	63.06	50.27	82	36.94	49.73	222
2013	133	59.91	46.53	89	40.09	53.47	222

**Fig. 1:**
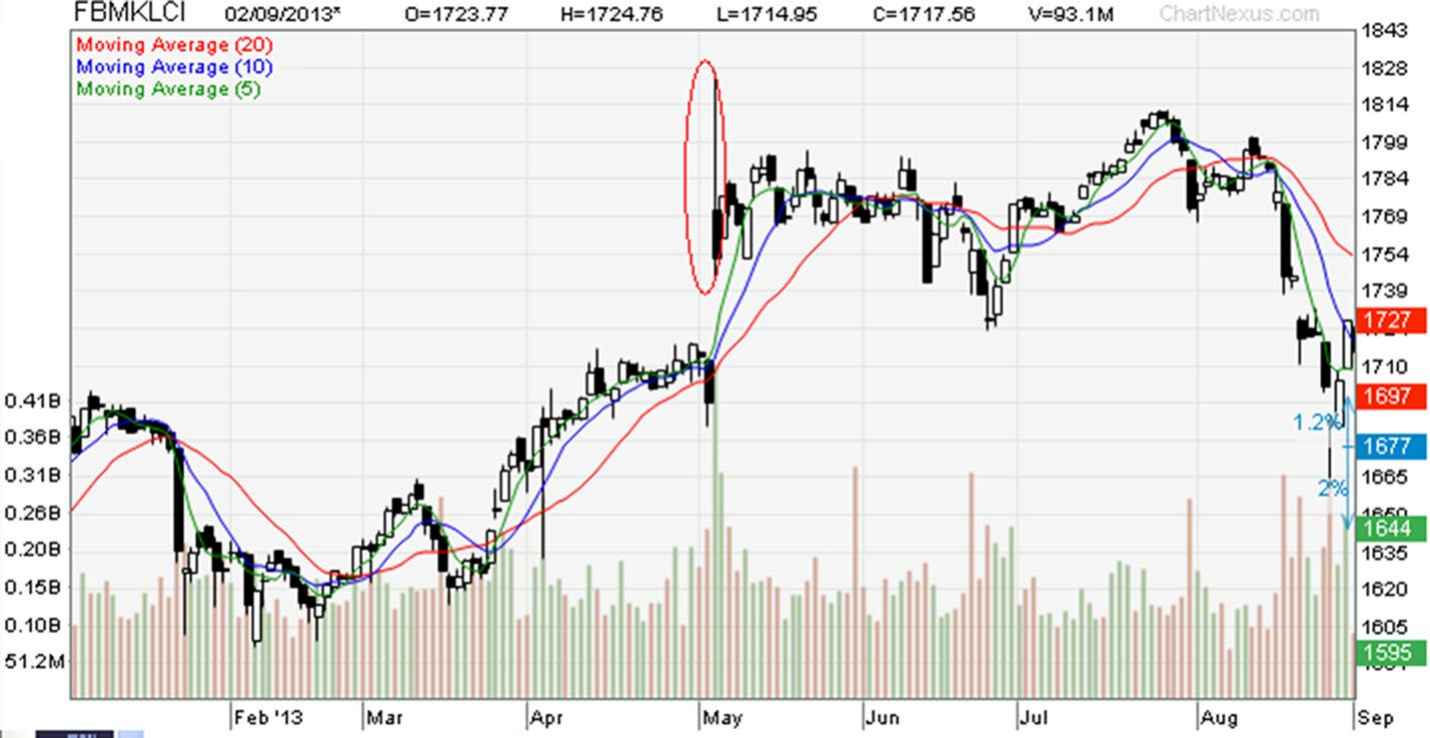
Snapshot of FMBKLCI daily performance before and After the 13th Malaysia general election (5th May 2013). *Source*: Courtesy of RHB Investment Bank Limited, Malaysia

After the most recent general election which was held on 5th May 2013, the FTSE Bursa Malaysia Kuala Lumpur Composite Index, abbreviated as FBMKLCI, hits the historical highest peak. Referring to Fig. 1, the daily index achieved a short term gain of 96.29 point by exhibiting a steady rising trend starting from a previous low of 1621.36 point on the 18th March 2013, to 1717.65 point on 30th April 2013. The Ragoff’s (1990) equilibrium political budget cycle theory may apply here to rationalize this trend.

However, the index dipped 22.88 points altogether in just two days prior to the election date. This is probably due to the reason that investors feared that the incumbent National Front government may loss its power to the opposition coalition (People’s Alliance), which gave the fiercest challenge ever to the former who has been ruling the country since its independent on 31 August 1957. Nonetheless, the index which was traded with substantially high daily volume lifted 62.52 points on the next trading day (6th May 2013) after the announcement of the outcome in the election night. In fact, the index registered a highest gain of 96.29 point in the middle of the intra-day trade on 6th May 2013. This suggests profitable intra-day trading after the event. Apparently, Bursa Malaysia investors’ confidence was boosted by the re-election of the incumbent government. In this backdrop of the most recent experience of the Malaysia stock market performance corresponding to general election, this study aims to investigate if there is any significant before-election-effect and after-election-effect on the FBMKLCI daily returns.^3^

## Data and empirical method

The daily FBMKLCI data set employed in this study was collected from *Datastream* and the election dates were obtained from the Electoral Commission of Malaysia.^4^ The sample period ranges from 1995 to 2013, which covers the most recent five general elections. The event dates were 25th April 1995 (Ninth General Election), 29th November 1999, 21st March 2004, 8th March 2008 and 5th May 2013 (Thirteen General Election). The percentage returns data for this study is calculated from the daily FBMKLCI.

The empirical model used in this study follows the regression-based approach conducted by Abidin et al. (2010), in line with the objective to investigate the returns of FBMKLCI before and after the elections. Nonetheless, it has no intention to investigate which parties might affect the returns of stock market index value since the existing ruling party is on the lead for more than half a century. Therefore, the modified equation is illustrated as follows:

1 $$ R_{t} = \, \beta_{0} + \, \beta_{1} B_{t} + \, \beta_{2} A_{t} + \, \varepsilon_{t} , $$

where, *R*_*t*_ = stock index return at time *t*; *B*_*t*_ = dummy variable that equals to one for N trading days before election and zero otherwise, (N = 15, 30, 60, 90); *A*_*t*_ = dummy variable that equals to one for N trading days after election and zero otherwise, (N = 15, 30, 60, 90); and *ε*_*t*_ = error term.

In this study stock index return is estimated as $R_{t} = 100 \times \left[ {ln\left( {I_{t} } \right) \, {-}ln(I_{t - 1} )} \right]$, where *I*_*t*_ and *I*_*t*−1_ are the FBMKLCI at time *t* and *t*-1 respectively and *ln* represents natural logarithm.

The implementation of Ordinary Least Squares (OLS) regression with dummy variable would allow us to determine whether the daily returns could be related to the general elections. This equation is estimated for 15, 30, 60, and 90 trading day windows to see the different effect of elections if any, on the returns of FBMKLCI at different time frames. If the estimated *β*_*1*_ is significant, it implies the general elections have significance effect on the daily returns before the event. Similarly, if the estimated *β*_*2*_ is significant, it implies the general elections have significance effect on the daily returns after the event. Conversely, there is no evidence of general election effect if none of them is statistically significant.

Apart from that, macroeconomic variables and US stock market returns are included in this study as control variables.^5^ As such, Eq. (1) is then extended to include the following variables: (1) US stock market return which is represented by the daily S&P 500 return (%); (2) Inflation rate (%), which is calculated from the daily world crude oil price; (3) Interest rate (%), which is the Malaysian daily average interbank deposit rate; (4) Unemployment rate (%), which is the monthly rate as the daily rate is unavailable; (5) Exchange Rate, which is the Malaysia ringgit per US dollar rate; (6) Percentage change in quarterly Gross Domestic Product as higher frequency data are unavailable; and (7) Malaysia stock market volatility. Following Opare (2012), stock market volatility (%) is estimated as $100 \times \left[ {ln\left( {H_{t} } \right){-}ln(L_{t} )} \right]$, where *H*_*t*_ and *L*_*t*_ denote the highest and lowest value of FBMKLCI at time *t*. This variable is taken as a proxy for political uncertainty, since past literature had documented that political uncertainty induced market volatility.^6^

## Results of analysis

The preliminary estimated results are summarised in Table 2. It is evident from Table 2 that there exists general election effect in the daily returns of FMBKLCI. This finding is true for every election under studied. However, the effect of each general election is different. In this respect, the magnitude and duration of the effect are distinct for different general election. Particularly, the Ninth General Election (25th April 1995) is statically associated to positive daily returns 60 trading days before and 60 trading days after the event. The estimated $\beta_{1}$ and $\beta_{2}$ are 0.38 and 0.36 respectively and they are statistically significant at 5% significance level for *N* = 60. It reveals that during the 60 trading days before the election, there was an additional average daily gain of 0.38% compared to ordinary days without general election. This rising trend continued after the election date with a slightly slower pace of additional 0.36% per trading day for 60 days compared to ordinary trading days.

**Table 2:** General election effect

*N*	1995	(9th GE)		1999	(10th GE)		2004	(11th GE)		2008	(12th GE)		2013	(13th GE)	
	*β*_0_	*β*_1_	*β*_2_	*β*_0_	*β*_1_	*β*_2_	*β*_0_	*β*_1_	*β*_2_	*β*_0_	*β*_1_	*β*_2_	*β*_0_	*β*_1_	*β*_2_
15	−0.09	0.15	0.35	0.06	0.11	0.31	0.07	0.13	−0.22	−0.02	−0.62*	−0.20	0.02	−0.07	0.26*
	−*1.00*	*0.45*	*1.03*	*0.56*	*0.27*	*0.75*	*1.26*	*0.60*	−*1.08*	−*0.20*	−*1.97*	−*0.63*	*0.55*	−*0.45*	*1.75*
30	−0.14	0.29	0.37	0.01	0.03	0.59*	0.06	0.32**	−0.31**	−0.05	−0.16	0.03	0.00	0.13	0.15
	−*1.48*	*1.18*	*1.49*	*0.08*	*0.11*	*1.94*	*1.05*	*2.14*	−*2.06*	−*0.57*	−*0.69*	*0.13*	−*0.05*	*1.19*	*1.40*
60	−0.23	0.38*	0.36*	−0.07	0.10	0.54**	0.09	0.16	−0.27*	−0.03	−0.14	−0.02	−0.01	0.08	0.12
	−*2.10*	*1.92*	*1.81*	−*0.52*	*0.43*	*2.22*	*1.27*	*1.33*	−*2.22*	−*0.25*	−*0.76*	−*0.10*	−*0.27*	*0.86*	*1.30*
90	−0.28	0.32	0.31	0.11	−0.22	0.15	0.17	−0.05	−0.25**	0.03	−0.07	−0.18	0.02	0.00	0.03
	−1.86	1.59	1.53	0.60	−0.89	0.58	1.80	−0.37	−2.04	0.19	−0.39	−0.95	0.30	0.05	0.32

GE denotes general election. The results are estimated from Eq. (1) based on a sample of 124 trading days before and 124 trading days after each general election. *N* denotes the size of the trading window. *β*_0_ represents the intercept term, where as *β*_1_ and *β*_2_ are coefficients of the dummy variables for *N* trading days before and after the election date respectively. The *t*-statistics are given in italic below the respective estimated coefficients

* and ** Significant at 10 and 5% significance level respectively. The significance of the estimated *β*_1_ and *β*_2_ implies there is before-election-effect and after-election-effect respectively

For the Tenth General Election (29th November 1999), it significantly corresponds to an extra 0.59 and 0.54% daily returns compared to ordinary trading days for 30 and 60 trading days respectively after the election date. A different scenario is observed for the Eleventh General Election (21st March 2004), where a positive effect (2.14%) is found in the 30 trading day-period before the election. However, the index reversed its upward trend after the election date such that when compared to ordinary trading days, the daily returns were reduced by 0.31, 0.27 and 0.25% during the first 30, 60 and 90 trading days respectively after the event. It is worth-mentioning that the effect of the Eleventh General Election could last up to 90 trading days, while it only lasted up to 60 trading after the earlier two events. In sharp contrast, the duration of effect for the following two general elections was shortened to 15 trading days only. Specifically, the Twelfth General Election (8th March 2008) exhibited a significant negative before-election-effect, while the Thirteen General Election (5th May 2013) showed a positive after-election-effect. According to Fama’s (1965) efficient market hypothesis, this finding may signify that the Bursa Malaysia is more information efficient for the last two general elections.

Other important findings from the analysis include: First, the stock market reaction towards general election was positive before the event (indicated by the positive value of the estimated $\beta_{1}$) for Ninth and Eleventh general election only. The stock market was significantly negative for the Twelfth General Election while there is no significant before-election-effect for the Thirteenth General Election. Second, the stock market reacted positively after the general election for the Ninth, Tenth, and Thirteenth General Election. In sharp contrast, the only negative stock market reaction is observed after the Eleventh General Election. There is no significant after-election-effect for the Twelfth General Election.

Having examine the effect of generation election on the Malaysian stock market return, the role of macroeconomic variables and market volatility in influencing the stock return is analysed. The regression results are summarised in Tables 3, 4, 5, 6 and 7 for 1995, 1999, 2004, 2008 and 2013 general elections respectively.

**Table 3:** The influence of macroeconomic variables and market volatility on the market return during 1995 general election

Variable	Day			
	15	30	60	90
Constant	0.01	−0.12	−0.24	−0.14
	*0.07*	−*0.64*	−*1.32*	−*0.59*
Before (*β*_1_)	0.38	0.46	0.53*	0.24
	*0.71*	*1.13*	*1.89*	*0.91*
After (*β*_2_)	0.25	0.40	0.47*	0.22
	*0.61*	*1.21*	*1.69*	*0.93*
US stock market return	3.30	4.21	3.28	3.54
	*0.24*	*0.30*	*0.24*	*0.25*
Inflation rate	−0.03	−0.02	−0.01	−0.02
	−*1.10*	−*0.89*	−*0.28*	−*0.63*
Interest rate	–	–	–	–
	–	–	–	–
Unemployment rate	–	–	–	–
	–	–	–	–
Exchange rate	0.10	0.12	0.17	0.07
	*0.81*	*1.03*	*1.48*	*0.69*
Gross domestic product	−0.03	−0.02	−0.02	−0.03
	−*1.23*	−*0.45*	−*0.60*	−*1.03*
Market volatility	−0.29	−0.26	−0.26	−0.26
	−*0.73*	−*0.67*	−*0.67*	−*0.66*

Before and after are dummy variables to capture the impact of election effect on stock market return. The *t*-statistics are given in italic below the respective estimated coefficients

* and ** Significant at 10 and 5% significance level respectively. The significance of the estimated *β*_1_ and *β*_2_ implies there is before-election-effect and after-election-effect respectively. Interest rate and unemployment rate data are unavailable for the year 1995 and so they are excluded in this estimation

**Table 4:** The influence of macroeconomic variables and market volatility on the market return during 1999 general election

Variable	Day			
	15	30	60	90
Constant	−0.56	−0.51	−0.16	−0.54
	−*1.54*	−*1.39*	−*0.36*	−*1.45*
Before (*β*_1_)	0.76	0.06	0.27	−0.42
	*1.23*	*0.15*	*0.90*	−*1.53*
After (*β*_2_)	−0.25	0.46	1.02	−0.12
	−*0.46*	*0.98*	*1.58*	−*0.32*
US stock market return	0.06	0.06	0.07	0.07
	*0.69*	*0.73*	*0.86*	*0.88*
Inflation rate	−0.01	−0.01	−0.01	−0.01
	−*0.71*	−*0.61*	−*0.59*	−*0.63*
Interest rate	0.03	0.03	0.03	0.03
	*1.17*	*1.16*	*1.21*	*1.19*
Unemployment rate	0.03	0.03	−0.04	0.04
	*1.3*	*0.96*	−*0.75*	*1.44*
Exchange rate	4.60	−0.34	0.81	−0.32
	*1.14*	−*0.09*	*0.29*	−*0.12*
Gross domestic product	0.12	0.10	−0.12	0.17*
	*1.25*	*0.96*	−*0.64*	*1.74*
Market Volatility	15.54	14.53	13.35	15.8
	*1.43*	*1.33*	*1.25*	*1.47*

Before and after are dummy variables to capture the impact of election effect on stock market return. The *t*-statistics are given in italic below the respective estimated coefficients

* and ** Significant at 10 and 5% significance level respectively. The significance of the estimated *β*_1_ and *β*_2_ implies there is before-election-effect and after-election-effect respectively

**Table 5:** The influence of macroeconomic variables and market volatility on the market return during 2004 general election

Variable	Day			
	15	30	60	90
Constant	0.02	−0.08	−0.07	0.02
	*0.07*	−*0.31*	−*0.27*	*0.06*
Before (*β*_1_)	0.47*	0.60**	0.27*	0.07
	*1.84*	*3.32*	*1.78*	*0.43*
After (*β*_2_)	0.08	−0.12	−0.06	−0.14
	*0.32*	−*0.49*	−*0.24*	−*0.78*
US stock market return	0.04	0.03	0.04	0.04
	*0.85*	*0.71*	*0.91*	*0.97*
Inflation rate	0.01	0.01	0.01	0.01
	*1.06*	*0.88*	*0.94*	*0.74*
Interest rate	−0.02	−0.01	−0.01	−0.01
	−*1.56*	−*0.76*	−*0.52*	−*0.12*
Unemployment rate	0.60	1.17**	0.47	0.54
	*1.20*	*2.17*	*0.95*	*1.03*
Exchange rate	−0.02	−0.04	−0.02	−0.02
	−*0.08*	−*0.21*	−*0.09*	−*0.07*
Gross domestic product	0.01	0.03	0.03	0.03
	*0.11*	*0.55*	*0.48*	*0.46*
Market volatility	−4.4	−4.96	−4.08	−3.97
	−*0.45*	−*0.52*	−*0.41*	−*0.40*

Before and after are dummy variables to capture the impact of election effect on stock market return. The *t*-statistics are given in italic below the respective estimated coefficients

* and ** Significant at 10 and 5% significance level respectively. The significance of the estimated *β*_1_ and *β*_2_ implies there is before-election-effect and after-election-effect respectively

**Table 6:** The influence of macroeconomic variables and market volatility on the market return during 2008 general election

Variable	Day			
	15	30	60	90
Constant	0.34	0.27	0.16	0.30
	*0.44*	*0.34*	*0.19*	*0.36*
Before (*β*_1_)	−0.60*	−0.14	−0.21	−0.09
	−*1.93*	−*0.57*	−*0.97*	−*0.42*
After (*β*_2_)	0.19	0.25	0.27	−0.16
	*0.51*	*0.79*	*0.60*	−*0.52*
US stock market return	0.07	0.07	0.06	0.07
	*1.08*	*1.11*	*1.04*	*1.14*
Inflation rate	−0.01	−0.01	−0.02	−0.01
	−*0.76*	−*0.59*	−*1.14*	−*0.30*
Interest rate	−0.08	−0.06	−0.08	−0.09
	−*0.15*	−*0.12*	−*0.15*	−*0.17*
Unemployment rate	0.03	0.03	0.03	0.04
	*0.72*	*0.64*	*0.70*	*0.74*
Exchange rate	−0.16**	−0.14**	−0.19**	−0.14**
	−*2.63*	−*2.15*	−*2.80*	−*2.12*
Gross domestic product	0.08	0.09	0.11	0.09
	*0.55*	*0.58*	*0.72*	*0.57*
Market volatility	−70.85**	−70.63**	−69.72**	−69.16**
	−*6.00*	−*6.13*	−*6.11*	−*6.03*

Before and after are dummy variables to capture the impact of election effect on stock market return. The *t*-statistics are given in italic below the respective estimated coefficients

* and ** Significant at 10 and 5% significance level respectively. The significance of the estimated *β*_1_ and *β*_2_ implies there is before-election-effect and after-election-effect respectively

**Table 7:** The influence of macroeconomic variables and market volatility on the market return during 2013 general election

Variable	Day			
	15	30	60	90
Constant	0.14*	0.09	0.07	0.01
	*1.85*	*1.15*	*0.66*	*0.07*
Before (*β*_1_)	−0.09	0.23	0.01	0.01
	−*0.49*	*1.35*	*0.05*	*0.05*
After (*β*_2_)	0.26	0.23*	0.25*	0.36**
	*1.41*	*1.71*	*1.97*	*2.16*
US stock market return	0.05	0.05	0.05	0.05
	*0.88*	*0.88*	*0.81*	*0.86*
Inflation rate	−0.02	−0.01	−0.03	−0.03*
	−*1.02*	−*0.46*	−*1.30*	−*1.68*
Interest rate	0.03	0.03	0.05*	0.07**
	*0.98*	*1.30*	*1.71*	*2.36*
Unemployment rate	0.02	0.03	0.02	0.02
	*0.50*	*0.69*	*0.41*	*0.55*
Exchange rate	0.03	0.03	0.03	0.03
	*0.67*	*0.70*	*0.65*	*0.66*
Gross domestic product	−0.02	−0.01	−0.02	−0.03
	−*0.65*	−*0.04*	−*0.61*	−*1.00*
Market volatility	−17.53**	−19.79**	−15.72**	−18.45**
	−*2.29*	−*2.54*	−*2.04*	−*2.42*

Before and after are dummy variable to capture the impact of election effect on stock market return. The *t*-statistics are given in italic below the respective estimated coefficients

* and ** Significant at 10 and 5% significance level respectively. The significance of the estimated *β*_1_ and *β*_2_ implies there is before-election-effect and after-election-effect respectively

It is could be seen from Table 3 that general election effect is present in 60 days trading window before and after the 1995 General Election. The estimated $\beta_{1}$ and $\beta_{2}$ are 0.53 and 0.47 respectively and they are statistically significant at 10% significance level for *N* = 60. It reveals that during the 60 trading days before the election, there was an additional average daily gain of 0.53% compared to ordinary days without general election. This rising trend continued after the election date with a slightly slower pace of additional 0.47% per trading day for 60 days compared to ordinary trading days. Such before-election-effect and after-election-effect are not observed in other trading period. Moreover, the macroeconomic variables and market volatility which are included as regressors play no significant role in influencing the stock market return. One the other hand, Table 4 shows that neither the 1999 General Election nor the control variables has any significance role in influencing stock market return, with one exception. The only exception occurs for *N* = 90, whereby percentage change in gross domestic product is found to have positive relation on the stock market return. This indicates that economic growth is the important concern of investors in around this election year. On quarter to quarter basis, this economic growth indicator was found to decline from 9.12% the second quarter in the election year, to 5.89% in the third quarter and then to 4.22% in the fourth quarter, in which the election took place. It further dropped to 1.68% 90 days, before it recovered to 4.82 and 6.92% respectively in the first quarter in the following year.

Table 5 shows that the 2004 General Election played a significance role in influencing the stock market return, for *N* = 15, 30 and 60 before election. The estimated *β*_*1*_ are 0.47, 0.60 and 0.27 respectively, indicating during 15, 30 and 60 trading days before the election, there was an additional average daily gain of 0.47, 0.60 and 0.27% compared to ordinary days without general election. As for control variables, only unemployment rate had significance impact on the market return for *N* = 30. The positive sign perhaps indicate that the market was positive on the newly re-elected government in dealing with unemployment issue. In fact, the unemployment stood at 3.8% in the first quarter of 2004, in which the election was held. It did actually gradually decrease and eventually fell to 3.1% 1 year after the election. Smales (2015), on the other hand, provides empirical evidence that percentage change in unemployment has significant positive impact on volatility of Australian stock market return However, unemployment rate had no impact on the stock market return for longer trading windows. Perhaps the impact had been fully priced-in 0n the stock return in the first 30 days trading window before the election.

As for the 2004 General Election, a negative and significant before-election-effect is reported for *N* = 15 in Table 6. Other than that, it had no significance effect on the stock market return. Notably, exchange rate and stock market volatility were found to have negative and significant influences on the stock market return. Kirui et al. (2014) also found negative impact of exchange rate on stock market return. Our findings could be due to the 2008 Global Finance Crisis, which began in 2007 when the rocketing home prices in the United States finally plummeted and henceforth affected the entire U.S. and overseas financial markets.

Table 7 shows that the 2013 General Election had resulted in positive and significant after-election-effect for *N* = 30, 60 and 90, which brought about 0.23, 0.25 and 0.36% of additional average daily return compared to ordinary days with no election. This may signal that the market received the election outcome well, in which the incumbent was re-given mandate to continue governing the country for another 5 years’ term. Additionally, interest rate, inflation rate and market volatility played significantly role in the stock return around the election year. This is consistent with previous studies that found stock market return are related to these variables and political uncertainties (see for instance, Papadamou et al. 2016; Smales 2015).

## Conclusion

Since its independence, Malaysia has undergone thirteen episodes of general election as of today. The National Front coalition managed to win all of them. However, during the last two episodes of general election, the competition among the National Front coalition and the People’s Alliance was so close that the chance of winning was 50–50. In particular, in the 2013 General Election, the opposition party actually had won 53.47% of vote but it managed to secure only 40.09% of the parliament seats. As such, the incumbent was once again re-elected to form the government.

Few researchers have put forward theories to hypothesize that political elections will have significant effect on the stock market. Previous studies using stock market data from the developed countries were able to support this hypothesis. In this conjunction, the current study finds significant before-election-effect and after-election-effect from the most recent general elections held in Malaysia. Preliminary analysis was conducted using Ordinary Least Squares regression model. The results obtained reveal that, out of the five general elections under studied, 40% of the time the stock market reacted positively before the elections, whereas 60% of the time the market reacted positively after the elections. For further analysis, the regression model is augmented with control variables. This study also manages to find evidence of general election effect even after the inclusion of macroeconomic variables and market volatility as control variables.

As for control variables, different subsets of macroeconomic variables are found to have significant role on stock market return depending on the market situation. For instance, during financial market turbulence in 2008, exchange rate played a significant role in negatively influencing the stock market return.

Notably, while market volatility which represents political uncertainty had no impact on stock market return on the general election years of 1995, 1999 and 2004, it did show its significance influence in the 2008 and 2013 election years. In these two episodes of general election, the incumbent National Front had been fiercely challenged by its opponent, the People’s Alliance. This is evident from the very close percentage of votes and percentage of seats obtained by both parties for these two elections. As a matter of fact, during the 2013 General Election, the People’s Alliance had secured more votes, but the National Front had won more seats. However, the winner was decided based on number of seats and thus during the incumbent National Front once again formed the government. On the other hand, in the earlier three episodes of general election, the National Front had won majority of the parliament seats (84.38, 76.68 and 90.41% of the seats, in 1995, 1999 and 2004 General Elections, respectively, see Table 1). Hence, it can be said that the stock market return was unaffected due to the calm atmosphere of the general election around the 1995, 1999 and 2004 General Elections.

Note that the 13th Parliament of Malaysia will automatically dissolve on 24 June 2018. Thus, the next Malaysia general election is around the corner as the leader of the incumbent government may opt to dissolve the parliament earlier to gain political advantage. In this respect, the major implication of these findings is that while investors may seek abnormal returns before and after the next general election, they will have to pay attention on the influence of macroeconomic variables on stock market return during the election year. As for future direction of study, interested reader may take up the election effect on the Malaysia stock market volatility, with reference to Smales (2014, 2015, 2016).

## Footnotes

1 See also the references therein for more studies on the impact of political election on stock market returns.

2 Federation of Malaya (now known as West Malaysia) gained its independence from the British on August 31, 1957. Together with the states of Sabah and Sarawak (East Malaysia), which were seperate British colonies, Federation of Malaysia was formed in July 22, 1963.

3 Numerous researches have been conducted to study stock market anomalies in Malaysia. These include Wong et al. (1990), Yong and Ibrahim (1999), Davidson and Peker (1996), Goh and Kok (2004), Chia et al. (2006), and Lean and Tan (2010). These studies investigate calendar anomalies like month-of-the-year, day-of-the-week, time-of-the-day, and seasonality.

4
http://www.spr.gov.my/.

5 Thanks to an anoymous referee who pointed out the importance of the inclusion of political uncertainty, macroeconomic variables and US stock market returns.

6 See for instance, Smales (2014, 2015, 2016), which construct measure of election uncertainty based on opinion poll and probability of succes of the incumbent party. Nonetheless, such data are unavailable for the case of Malaysia.
